# Infinite product expansion of the Fokker–Planck equation with steady-state solution

**DOI:** 10.1098/rspa.2015.0084

**Published:** 2015-07-08

**Authors:** R. J. Martin, R. V. Craster, M. J. Kearney

**Affiliations:** 1 Apollo Global Management International LLP, 25 St George Street, London W1S 1FS, UK; 2 Department of Mathematics, Imperial College London, South Kensington, London SW7 2AZ, UK; 3 Faculty of Engineering and Physical Sciences, University of Surrey, Guildford GU2 7XH, UK

**Keywords:** Ornstein–Uhlenbeck process, infinite product, asymptotics, diffusion, mathematical finance

## Abstract

We present an analytical technique for solving Fokker–Planck equations that have a steady-state solution by representing the solution as an infinite product rather than, as usual, an infinite sum. This method has many advantages: automatically ensuring positivity of the resulting approximation, and by design exactly matching both the short- and long-term behaviour. The efficacy of the technique is demonstrated via comparisons with computations of typical examples.

## 1. Introduction

### (a) Objectives

In this paper, we propose a novel analytical procedure for solving diffusion equations of the form

1.1 $$\begin{array}{rl} \frac{\partial f}{\partial \tau } & =-\frac{\partial \;}{\partial y}[A(y)f]+\frac{\partial^{2}f}{\partial y^{2}}\equiv L^{†}f\\ \text{and}\;\;f(0,y) & =\delta (y-Y_{0}), \end{array}\}$$

where the steady-state solution is well defined, i.e. a normalizable probability density (we call this the ‘stable’ case):

$$\int_{-\infty }^{\infty }\exp\left(\int_{0}^{y}A(\eta )\;d\eta \right)dy<\infty .$$

Generally, this partial differential equation (PDE) describes diffusion in the presence of a potential. For example, if it governs the concentration of diffusing charged ions in an electrostatic potential well it is generally referred to as the Nernst–Planck equation [1]. If the potential is not quadratic then one has (1.1) with *A* nonlinear and the equation is analytically intractable; techniques are therefore required to deal with it so that one does not have to fall back on full numerical solutions of the PDE.

More often, it arises as the forward (Fokker–Planck, FP) equation associated with the mean-reverting diffusion process

1.2 $$dY_{\tau }=A(Y_{\tau })\;d\tau +\sqrt{2}\;dW_{\tau }$$

in dimensionless form.

It is perhaps worth clarifying what we mean by ‘nonlinear diffusion’ as the term is ambiguous. A *diffusion process* such as (1.2) is said to be nonlinear if the drift term is nonlinear or the volatility term is non-constant; hence whenever *A* is nonlinear we have a nonlinear diffusion *process*. But the FP equation (1.1) that arises from it is always a *linear diffusion equation* (linear in *f*). That does not mean, however, that it is easy to solve. What is unusual about this paper is that we will be modifying (1.1) through a nonlinear change of the dependent variable *f* so that the diffusion equation does become nonlinear, and our thesis is that this nonlinear PDE is actually easier to handle than its linear counterpart.

In general, (1.2) is an important model of physical phenomena such as electronic noise and kinetics [2], electronic circuits with nonlinear resistance [3], and systems with overdamped Langevin dynamics [4] or from nonlinear friction [5,6]. Another example of application has been a model of variability of chemical concentration in ice-core data as a proxy of climate variability [7], where it is necessary to model large excursions. If *A*(*y*)=−*y*, we are left with the familiar Ornstein–Uhlenbeck (OU) process [8], or Langevin equation [9], but the nonlinear examples have *A*(*y*)≠−*y* and these require attention.

An analytical approximation to a PDE has considerable practical value aside from providing an immediate insight into the form of the PDE's solution as there is a considerable improvement in computation speed over the standard numerical grid-based methods. This is particularly true in the context of statistical signal processing techniques such as Markov chain Monte Carlo [10], where one wishes to sample from the distribution of *Y*_*t*_2__ given *Y*_*t*_1__, for *t*_2_>*t*_1_; knowing the density, one can sample using the rejection method [11]. Then an approximate solution that captures the short- and long-term behaviour is considerably more practical than having to regenerate the solution by numerically solving the PDE at each step. The time saved in that calculation can then be spent on running more Monte Carlo simulations. In fact, we suggest that even our leading-order term (3.23) is sufficiently accurate for the vast majority of practical applications, whatever the discipline.

Although there are physics applications of (1.2), as we have said, in fact it was mathematical finance that provided the main impetus for this research. The process (1.2) is a model of mean reversion and in the case when *A*(*y*)=−*y* it is the OU process which in dimensional coordinates is

1.3 $$dX_{t}=-\kappa X_{t}\;dt+\sigma \;dW_{t}.$$

But this has an important disadvantage, in that its steady-state distribution is Normal, so that large excursions are very unlikely in the model. Despite the fact that this has been known for years, the Normal distribution is still used in risk management in areas where it should not be. For example, investigation of JP Morgan Chase's ‘London whale’ trading losses, estimated at at least $5bn, shows that the Normal distribution function was used to transform a number of standard deviations into a loss probability even at high levels of confidence.^1^ Before that, the demise of Long Term Capital Management (LTCM) in 1998 can be directly attributed to over-leveraged ‘convergence trades’ [13]. In reality, the following two mechanisms occur when the market is far from equilibrium, and these cause such excursions to occur much more often than in the simple OU model.

**Figure 1.**
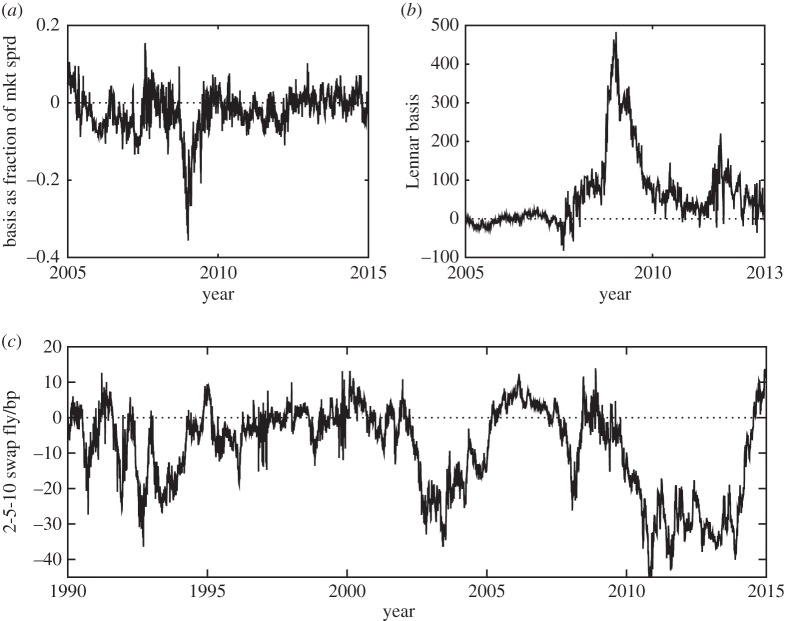
Basis examples: (*a*) Itraxx Main index basis history (market minus intrinsic level, divided by market level). (*b*) Corporate bond (Lennar 5.5% 14s) basis history. These are fitted quite well by (1.4). Relative value examples: (*c*) USD swap fly history, which is better fitted by (1.6).

The first mechanism is that the volatility is likely to be higher. This is seen in the so-called basis risks, in which the price difference between two closely related financial instruments should be zero: in times of market stress, when the distance from equilibrium is large, market liquidity is lower and the volatility higher, so large excursions become likely. One recent example is the so-called credit default swap (CDS) index basis, which is the difference between the index level of a CDS index contract and the level implied by its constituents, which should theoretically be nearly zero (see [14] for a general discussion on this). Another is the CDS-bond basis during the 2007–2009 financial crisis, where the credit spread of credit-risky cash bonds deviated violently from the level implied by the CDS market (e.g. [15], fig. 1, [16], fig. 1). The effect is clear in figure 1*a*,*b*, with pronounced departure from the mean during the 2007–2009 financial crisis. A convenient formulation increasing the volatility away from equilibrium is

1.4 $$dX_{t}=-\kappa X_{t}\;dt+\sigma \sqrt{1+\gamma^{2}X_{t}^{2}}\;dW_{t}.$$

Models such as this, in which the volatility depends deterministically on the spot price *X*_*t*_ and/or time, are called local volatility models (as distinct from stochastic volatility models; e.g. [17]). In fact (1.4) is precisely the same recipe as suggested in the aforementioned climate change paper [7]. It is also encountered as one of the Pearson–Wong diffusions [18,19] but has received less attention than the more commonly encountered OU and square-root processes also in that class, because it is less analytically tractable.

The second mechanism is that the reversion speed may decline. This is seen in examples where there is no strict requirement that prices must converge, and arises if the market volume directed towards a convergence bet declines, even if the total liquidity remains the same. An example is figure 1*c*, which shows the deviation between interest swap rates of three different tenors; this is the so-called ‘butterfly’ trade (e.g. [20]). We calculate the 5Y rate minus the weighted average, weights 0.3 and 0.7, respectively, of the 2Y and 10Y rates, after which the 10-year exponentially weighted moving average is subtracted so as to take out the long-term average; not the length of the excursion from 2009 (post-crisis) onwards. This second mechanism is captured by the following models:

1.5 $$dX_{t}=-\frac{\kappa X_{t}}{\sqrt{1+\gamma^{2}X_{t}^{2}}}\;dt+\sigma \;dW_{t}$$

and

1.6 $$dX_{t}=-\frac{\kappa X_{t}}{1+\gamma^{2}X_{t}^{2}}\;dt+\sigma \;dW_{t},$$

and also by (1.4) after transformation by $\gamma X=\sinh\hat{\gamma }Y$, $\hat{\gamma }=\gamma \sigma /\sqrt{2\kappa }$, giving

1.7 $$dY_{t}=-\frac{(1+{\hat{\gamma }}^{2})\kappa }{\hat{\gamma }}\tanh(\hat{\gamma }Y_{t})\;dt+\sqrt{2\kappa }\;dW_{t}.$$

**Figure 2.**
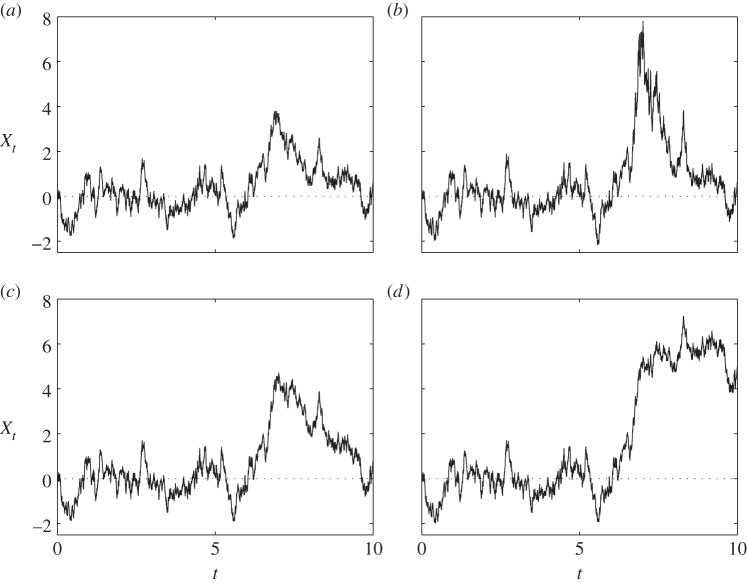
Typical realizations: (*a*) OU, i.e. *γ*=0; (*b*–*d*) equations (1.4)–(1.6) with $\gamma =\frac{1}{2}$, i.e. *ν*=5. (Using the same excitation process *W*_*t*_ each time; *σ*=2, *κ*=2, *ν*=1+2*κ*/*γ*^2^*σ*^2^.) Note the visual similarity between (*b*) and figure 1*a*,*b*; also between (*d*) and figure 1*c*.

The same effects can also easily be seen in simulation (figure 2*a*–*d*).

We have distinguished two mechanisms, but although the distinction is important for motivating an underlying model, it is unimportant to the issue of solving the forward equation. Indeed, as we just did with (1.4)⇝(1.7), we can transform the process to arrange for the volatility to be constant and thereby work with the canonical form (1.2). Examples (1.5) and (1.6) simply require a trivial rescaling $Y=X/\sqrt{\sigma^{2}/2\kappa }$; also *τ*=*κt*. In general, the transformation *Y* =*η*(*X*) with $\eta (x)=\int dx/\sigma_{X}(x)$ does this, provided *σ*_*X*_ is non-zero. The canonical forms are therefore (where we have non-dimensionalized (1.5) and (1.6) to use $Y=X/\sqrt{\sigma^{2}/2\kappa }$ rather than *X*)

$$\begin{array}{rl} \left(1.5):\;A(y\right) & =-\frac{y}{\sqrt{1+{\hat{\gamma }}^{2}y^{2}}}\\ \left(1.6):\;A(y\right) & =-\frac{y}{1+{\hat{\gamma }}^{2}y^{2}}\\ \left(1.7):\;A(y\right) & =-\frac{1+{\hat{\gamma }}^{2}}{\hat{\gamma }}\tanh\hat{\gamma }y \end{array}$$

and the corresponding invariant densities are

$$\begin{array}{rl} \left(1.4):\;f_{X}(\infty ,x\right) & =\frac{\gamma {\left(1+\gamma^{2}x^{2}\right)}^{-(\nu +1)/2}}{B(\nu /2,1/2)}\\ \left(1.5):\;f_{Y}(\infty ,y\right) & =\frac{\hat{\gamma }}{2K_{1}(\nu -1)}\cdot \exp\left(-(\nu -1)\sqrt{1+{\hat{\gamma }}^{2}y^{2}}\right)\\ \left(1.6):\;f_{Y}(\infty ,y\right) & =\frac{\hat{\gamma }{\left(1+{\hat{\gamma }}^{2}y^{2}\right)}^{-(\nu -1)/2}}{B((\nu -2)/2,1/2)}\\ \left(1.7):\;f_{Y}(\infty ,y\right) & =\frac{\hat{\gamma }{\left(\cosh\hat{\gamma }y\right)}^{-\nu }}{B(\nu /2,1/2)} \end{array}$$

(B and *K*_*ν*_ denoting as usual the Beta function and the modified Bessel function of the second kind; see [21]) and throughout

$$\hat{\gamma }=\gamma \sqrt{\frac{\sigma^{2}}{2\kappa }};\;\nu ={\hat{\gamma }}^{-2}+1$$

so that the non-dimensional parameter $\hat{\gamma }$ or *ν* measures the deviation from the OU model ($\hat{\gamma }=0$, ν=∞).

In each case, the density is fatter tailed than Gaussian. In (1.6), the density is fatter tailed than in (1.5) because the reversion disappears completely as the diffusion process moves far from equilibrium, and so it is left to wander around aimlessly.

There is a reasonable amount of similarity, at least visually, between figures 2*b* and 1*a*,*b*. We therefore tested the model (1.4), using the standard likelihood-ratio test, with the null hypothesis $H_{0}:\hat{\gamma }=0$ against the alternative $H_{1}:\hat{\gamma }>0$. The method yields a maximum-likelihood estimate for the parameter in question and an estimate of the relative likelihood of *H*_0_ versus *H*_1_ given the data (the *p*-value). For figure 1*a*, we found *ν*=3.8 with *p*-value 1×10^−8^, and for figure 1*b* we found *ν*=3.2 with *p*-value 1×10^−32^. The rejection of the basic OU model is unsurprising, given the huge excursions from equilibrium in both datasets. Similarly, figure 2*d* bears a resemblance to figure 1*c*. Testing (1.6) in the same way on figure 1*c*, we found *ν*=4.4 with *p*-value 5×10^−6^. This suggests that (1.6) is preferable to the simple OU model. (For the purposes of risk management, it is clearly prudent to use any of (1.5)–(1.7) in place of the basic OU, even if there is no firm statistical evidence.)

We round off this section by returning to physics, with an example that is considerably further from the OU process, qualitatively, at least in the sense of not being in a one-parameter deformation of the OU model. This is the double-well potential, where the invariant density is bimodal. This has been studied by Caroli *et al.* [22], who used the Wentzel–Kramers–Brillouin (WKB) approximation to study the behaviour in different time regimes; here we shall demonstrate a method of solution valid on all time scales simultaneously. For the sake of concreteness, we confine ourselves to a particular example

**Figure 3.**
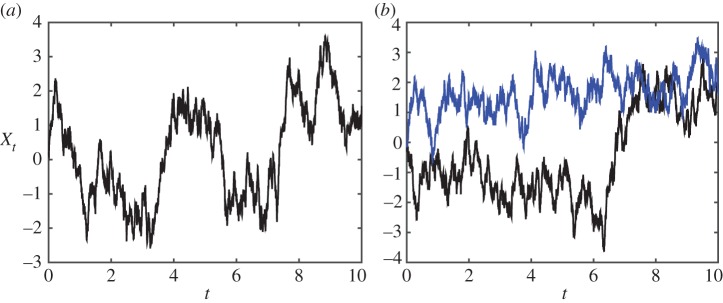
Simulations of the Ito process described by the double-well example (1.8) with *σ*=2, *κ*=2, *c*_1_=4, $c_{2}^{2}=2$ and *τ*=*κt*.

1.8 $$dX_{t}=\kappa \left(-x+\frac{c_{1}x}{1+c_{2}^{2}x^{2}}\right)dt+\sigma \;dW_{t},$$

where we require *c*_1_>1 (so that there are two wells), *c*_2_≠0 (for asymptotic stability). Figure 3 shows typical realizations of the corresponding Ito process, for *σ*=2, *κ*=2, *c*_1_=4, $c_{2}^{2}=2$, *τ*=*κt*. In some realizations such as figure 3*a* the process hops from one well to the other (these are centred at $X=\pm \sqrt{\frac{3}{2}}$); but it can spend a long time in one well (figure 3*b*). In non-dimensional form, this is

1.9 $$A(y)=-y+\frac{4y}{1+2y^{2}},\;f_{Y}(\infty ,y)=\frac{1}{3\sqrt{2\pi }}(1+2y^{2})\;e^{-y^{2}/2};$$

in general, $f_{Y}(\infty ,y)=\text{Gaussian}\times \text{polynomial}$, with the polynomial factorizable as a product of strictly positive quadratics, is a form that will construct multi-modal examples of arbitrary complexity.

### (b) Infinite products

The main thrust of this paper is that one benefits significantly from studying the logarithmic derivative, in the spatial direction, of the solution. An immediate advantage is that upon integration and re-exponentiation a positive solution must be obtained (there is still a time-dependent normalizing factor to obtain, but as we show later it is easy to ensure that this is positive).

Next, the logarithmic derivative is an algebraically simpler construction. Indeed, for the OU model one has

1.10 $$f_{X\;|\;X_{0}}(t,x)=\frac{1}{\sqrt{2\pi \xi^{2}(1-e^{-2\kappa t})}}\exp\left(-\frac{{\left(x-X_{0}\;e^{-\kappa t}\right)}^{2}}{2\xi^{2}(1-e^{-2\kappa t})}\right),\;\xi =\frac{\sigma }{\sqrt{2\kappa }}$$

whereas

$$-\frac{\partial \;}{\partial x}\ln f_{X\;|\;X_{0}}(t,x)=\frac{x-X_{0}\;e^{-2\kappa t}}{\xi^{2}(1-e^{-2\kappa t})}.$$

(In the case of an arithmetic Brownian motion with no reversion and starting from the origin, this would simply read *x*/*σ*^2^*t*.) We wish to replicate this exactly when *A* is linear, and this solution is a starting point in our analysis. Note that the form of (1.10) and the stochastic representation of the OU process as a scaled time-transformed Wiener process [23]

$$X_{t}=X_{0}\;e^{-\kappa t}+\xi \;e^{-\kappa t}W_{e^{2\kappa t}-1}$$

suggest a substitution *q*=e^−2*κt*^, and indeed we use this as an ansatz for mean-reverting processes in general.

The next issue is that we want a technique in which short- and long-time behaviour can be specified explicitly, and that these be replicated. This is because the former must be a Gaussian distribution as *t*→0, while the latter is the already-identified invariant density. Thus arrives the notion of an infinite product expansion, in the sense of writing the logarithmic derivative as the sum of a term that replicates the short-time behaviour, another that replicates the long-time behaviour, and a series that corrects the middle.

The infinite product is a significant departure from ‘standard’ techniques for solving parabolic PDEs; these have in common that they are all infinite *sums*. Three such are: spectral methods (Fourier transformation in the spatial coordinate [24]); orthogonal expansions (expand the spatial dependence as a time-weighted sum of eigenfunctions of the infinitesimal generator); and Laplace transformation in time. All suffer from the problem that it is difficult to represent the initial condition effectively. As a case in point, take the Mehler series expansion [25], in terms of Hermite polynomials He_*r*_(*x*), to the OU PDE:

1.11 $$f_{X\;|\;X_{0}}(t,x)=\frac{\exp(-x^{2}/2\xi^{2})}{\xi \sqrt{2\pi }}\sum_{r=0}^{\infty }\frac{e^{-r\kappa t}}{r!}{\mathrm{He}}_{r}\left(\frac{X_{0}}{\xi }\right){\mathrm{He}}_{r}\left(\frac{x}{\xi }\right),\;\xi =\frac{\sigma }{\sqrt{2\kappa }}.$$

**Figure 4.**
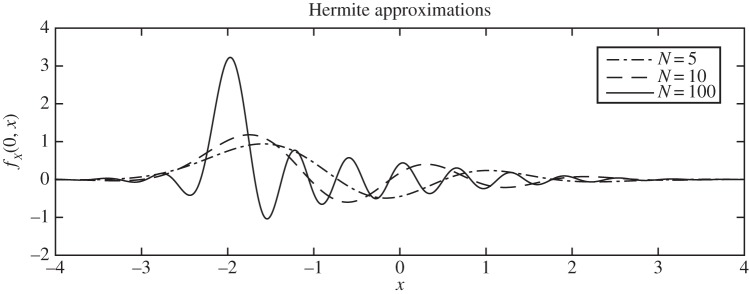
The truncation of the Mehler series expansion (1.11) of the OU solution is oscillatory, as shown here for *x* varying and *t*=0 for varying truncation of the series to *N* terms. (Parameters: *X*_0_=−2, *ξ*=1, $\kappa =\frac{1}{2}$.)

For *κt*≪1, it is clear from the sum that the convergence is very slow, because the basis functions, which are oscillatory, are required as *t*→0 to sum to give a delta function; truncation therefore generates oscillatory artefacts. Figure 4 shows the situation for *t*=0; indeed, the truncated Mehler series (taking only terms 0≤*r*<*N*) can be approximated around the spike *x*=*X*_0_ by the sinc function

$$\frac{\sin(\sqrt{N}(x-X_{0})/\xi )}{\pi (x-X_{0})}.$$

This behaviour is not specific to the OU process, and the slowness of convergence and the concomitant Gibbs phenomenon are a consequence of the Riemann–Lebesgue lemma. Yet for an infinite *product*, it is easy to make the short-time solution zero at all *x*≠*X*_0_, and hence like a delta function. One simply arranges for one of the terms in the product to be zero for *t*=0, *x*≠*X*_0_, and then it does not matter what the rest of the terms are: so another can be responsible for modelling the long-term behaviour, and the others for the middle-time region—as we intimated above. Even if one does not bother to correct the ‘middle-time’ behaviour, the results are remarkably good: this is seen later in equation (3.23) and in §3e and accompanying figures.

Probabilistically, infinite products have the attractive interpretation that each term in the product is the Radon–Nikodym derivative of the successive partial products. In statistical physics, the entropy is an important concept, and an infinite product represents this much more effectively than does a sum; from the point of view of an approximation, it is clearly disastrous to have a negative probability density anywhere. Note also that under a change of ‘spatial coordinate’ *X*, to *Y* =*η*(*X*) say, a multiplicative factor of |*η*′(*X*)| enters, so multiplicative representations are preserved under change of variable (because the Jacobian is just another term in the product) in a way that additive ones are not. Accordingly, it is quite incorrect to view an infinite product as a minor variant on an infinite sum: rather, we argue, the infinite product is fundamentally different and also a clearer way of thinking about the problem. On the other hand, the introduction of a logarithm causes a quadratic nonlinearity to appear in the FP equation, making the algebra considerably more difficult. In fact, the series expansion of its solution generates a quadratic recurrence of self-convolutive type somewhat analogous to that discussed in [26].

## 2. Preliminaries

By substituting $f_{X}(t,x)=g_{X}(t,x)f_{X}(\infty ,x)$ or $f_{Y}(t,y)=g_{Y}(t,y)f_{Y}(\infty ,y)$ and working with *g* instead, we arrive at the adjoint FP equation. This is similar to (1.1):

2.1 $$\frac{\partial g_{Y}}{\partial \tau }=A(y)\frac{\partial g_{Y}}{\partial y}+\frac{\partial^{2}g_{Y}}{\partial y^{2}}$$

and is also known as the backward equation (a term that we do not use here, because the equation arises as a forward equation: *τ* represents calendar time). This development is useful because we are going to ensure that, when we approximate *g*_*Y*_ (and hence *g*_*X*_ also), its long-term asymptote is unity. By so doing, we ensure that the approximated *f* tends to the correct invariant density.

As motivated in the Introduction, we deal with the logarithmic derivative of *g*_*Y*_ rather than with *g*_*Y*_ itself. We therefore introduce a function *h*_*Y*_,

$$h_{Y}(\tau ,y)=-\frac{\partial \;}{\partial y}\ln g_{Y}(\tau ,y),$$

so that *h*_*Y*_ satisfies an equation analogous to Burgers':

2.2 $$\frac{\partial h_{Y}}{\partial \tau }=\frac{\partial \;}{\partial y}\left\{A(y)h_{Y}+\frac{\partial h_{Y}}{\partial y}-h_{Y}^{2}\right\}.$$

Conventionally, the Hopf–Cole transformation [27] is used to convert the (nonlinear) Burgers equation into the (linear) diffusion equation, which is regarded as being more easily analysed. We are doing it backwards here, which may seem perverse: but note that this is also how the WKB approximation works [28], so the logarithmic transformation to a nonlinear equation has a well-established precedent.

The apparent similarity to the WKB expansion is worth discussing briefly. The similarity in form of the recursion is similar, as the logarithmic-derivative transformation also gives rise to what is, in essence, a Riccati equation. However, WKB is most naturally applied to problems in which the coefficient of the second derivative—here, the volatility (noise) term—is small. It gives rise to a singular expansion, because for zero noise the order of the differential equation reduces from two to one. That is not what we do: our approach is to take the OU solution for *h*_*Y*_(*τ*,*y*) and add terms to correct it. Thus whereas WKB expands the solution around a deterministic system, our techniques expand it around a known stochastic one. If the noise is very small, WKB may be more effective, but we have not observed a general failure of our methods in that limit.

## 3. Infinite product expansion

### (a) Solution of nonlinear partial differential equation by series expansion

We now seek a series expansion as a solution of (2.2). In the regular OU case, *A*(*y*)=−*θy*, we have

$$h_{Y}(\tau ,y)=\frac{\theta qy-\theta \sqrt{q}Y_{0}}{1-q},\;q=e^{-2\theta \tau }.$$

Now for *general* *A*, we still have a diffusion starting from *Y*_0_, in which the distribution's variance initially grows with time as 2*τ*, so if we expand around *τ*=0, i.e. *q*=1, in powers of (1−*q*), we are led to consider a development

$$h_{Y}(\tau ,y)=\frac{\theta (y-Y_{0})+o(1-q)}{1-q},\;q=e^{-2\theta \tau },$$

where *θ* is an *arbitrary* constant, and will be set later. This can also be established by dominant balance in (2.2), as on the RHS the $h_{Y}^{2}$ term predominates as *τ*→0. The basic OU solution is corrected by a series expansion thus:

3.1 $$h_{Y}(\tau ,y)=\frac{\theta qy-\theta \sqrt{q}Y_{0}}{1-q}+\sum_{r=0}^{N-1}q{\left(1-q\right)}^{r}b_{r}(y)+R_{N}(\tau ,y)$$

with *R*_*N*_ denoting the remainder. Note that *N*=0, which we call the leading-order approximation, means that *no* (*b*_*r*_) terms enter. Many of the manipulations in the first part of this section serve to exchange terms of the form 1, $\sqrt{q}$, *q*, the point simply being that any two of these differ by *o*(1−*q*) in the vicinity of *q*=1.

The form of (3.1) is very important. Any truncation of the series must vanish as τ→∞, which is what we need, because all terms contain a factor of *q* or $\sqrt{q}$. Note also that the truncated error is uniformly bounded in *τ*, because the expansion is in *q*(1−*q*)^*r*^ and *q* lies in the compact interval [0,1]. Thus, for each *N* and each *y* the error in *h*_*Y*_(*τ*,*y*) is bounded in 0≤τ≤∞. Such uniformity is obviously not shared by, for example, a series expansion in powers of *τ*, which is why we dismissed that idea out of hand.

The functions (*b*_*r*_), and the remainder, *are dependent on* *Y*_0_, and should therefore be thought of as *b*_*r*_(*y* | *Y*_0_): for reasons of conciseness we just write *b*_*r*_(*y*) or just *b*_*r*_, and when we write *b*_*r*_′ it means that the *y*-dependence, not the *Y*_0_-dependence, is being differentiated.

We are shortly going to equate coefficients of powers of (1−*q*), and for that reason the $\sqrt{q}$ term in (3.1) is unwelcome; we therefore exchange $\sqrt{q}$ for *q* plus a Taylor series around *q*=1. Writing $\sqrt{q}={\left(1-(1-q)\right)}^{1/2}$ and using the binomial theorem, we find

3.2 $$\frac{\sqrt{q}-q}{1-q}\equiv \sum_{r=0}^{\infty }q{\left(1-q\right)}^{r}\delta_{r},\;\delta_{r}=\frac{1}{2^{2r+1}}\left(\begin{array}{c} 2r+1\\ r \end{array}\right),\;|1-q|<1.$$

Note also that $\delta_{0}=\frac{1}{2}$ and $(r+2)\delta_{r+1}=(r+\frac{3}{2})\delta_{r}$, which is needed later. Thus, the revised series is

3.3 $$h_{Y}(\tau ,y)=\frac{\theta q(y-Y_{0})}{1-q}+\sum_{r=0}^{N-1}q{\left(1-q\right)}^{r}(b_{r}(y)-\theta \delta_{r}Y_{0})+R_{N}^{♯}(\tau ,y)$$

with a modified remainder term (the term $R_{N}^{♯}$ now contains all of *R*_*N*_, plus the *r*≥*N* part of the infinite sum in (3.2)). The next step is to compute the (*b*_*r*_) by comparing coefficients in *q*(1−*q*)^*r*^. Note that the recurrence we are about to derive for the (*b*_*r*_) will be obtained from (3.3), but for computational purposes we use (3.1), of course omitting the remainder term.

It is convenient to write *b*_−1_(*y*)≡*θ*(*y*−*Y*_0_) so that the first term can be absorbed into the summations, and also *δ*_−1_=0. We then substitute (3.3) into

3.4 $$2\theta q\frac{\partial h}{\partial q}+\frac{\partial \;}{\partial y}\left\{Ah+\frac{\partial h}{\partial y}-h^{2}\right\}=0$$

and equate terms in *q*(1−*q*)^*r*^. (Note that terms of the form *q*^2^(1−*q*)^*r*^, which arise from the quadratic term in (3.4), have to be written as *q*(1−*q*)^*r*^−*q*(1−*q*)^*r*+1^.) This gives

$$\begin{array}{rl} & 2(r+1)(b_{r}-\theta \delta_{r}Y_{0}-b_{r+1}+\theta \delta_{r+1}Y_{0})+{\left(Ab_{r}-A\theta \delta_{r}Y_{0}\right)}^{\prime }+b_{r}^{\prime \prime }\\ & \;-2\sum_{j=-1}^{r+1}b_{j}^{\prime }(b_{r-j}-\theta \delta_{r-j}Y_{0})+2\sum_{j=-1}^{r}b_{j}^{\prime }(b_{r-1-j}-\theta \delta_{r-1-j}Y_{0})=0. \end{array}$$

For *r*=−1, we deduce

$${\left((y-Y_{0})(b_{0}-\frac{1}{2}\theta Y_{0})\right)}^{\prime }=\frac{1}{2}{\left((y-Y_{0})A\right)}^{\prime }+\theta (y-Y_{0}),$$

so

3.5 b0=12(A+θy)+const.y−Y0

with the last part being discarded as it is singular at *y*=*Y*_0_. Note that *b*_0_ happens not to depend on *Y*_0_, but all the others generally will. Thus, for *r*≥0,

3.6 $$\begin{array}{rl} & \theta (y-Y_{0})b_{r+1}^{\prime }+\theta (r+2)b_{r+1}\\ & \;=\theta \left(r+\frac{3}{2}\right)b_{r}+\left(\theta (y-Y_{0})+\frac{1}{2}A\right)b_{r}^{\prime }+\frac{1}{2}b_{r}^{\prime \prime }+\sum_{j=1}^{r}(b_{r-j}-\theta \delta_{r-j}Y_{0})(b_{j-1}^{\prime }-b_{j}^{\prime }). \end{array}$$

(The summation is void if *r*<1.) Accordingly,

3.7 br+1(y)=θ−1(y−Y0)−r−2∫Y0y(η−Y0)r+1[(θ(y−Y0)+12A(η))br′(η)+12br″(η)+∑j=1r(br−j(η)−θδr−jY0)(bj−1′(η)−bj′(η))θ(r+32)br(η)+(θ(y−Y0)+12A(η))br′(η)+12br″(η)+∑j=1r(br−j(η)−θδr−jY0)(bj−1′(η)−bj′(η))]dη.

When *y*=*Y*_0_, this is just 1/*θ*(*r*+2)×r.h.s.(3.6). The lower limit in the integral must be *Y*_0_ as otherwise *b*_*r*+1_ becomes singular at *y*=*Y*_0_ in a similar manner to (3.5). Successive terms may be extracted recursively; and fortunately, just as with the WKB expansion, the (differential) equation for *b*_*r*+1_ is first-order *linear*, even though it is nonlinear in the ‘known’ terms ${\left(b_{j}\right)}_{j=0}^{r}$. Thus, the recurrence involves no more than a succession of integrals.

### (b) The parameter *θ*

The parameter *θ* allows us to expand the FP equation at hand around that of any of a one-parameter family of OU equations in which *θ* governs the reversion speed. We want to know what is the best *θ* to use for any given *A*, and intuitively it is clear that one should use a *θ* that best approximates, in some sense, the (*y*-dependent) rate of mean reversion that *A* gives. In so doing, we will have matched not just the short- and long-time behaviour of the function *h*, but also the rate of transition from one regime to the other.

The bound states of (1.1) are the normalizable eigenfunctions of $L^{†}$. In the OU case with *Y*_0_=0, it is plain from the Mehler expansion (1.11) that the eigenfunctions *ψ*_*r*_(*y*) and associated eigenvalues λ_*r*_ are

$$\psi_{r}(y)=\sqrt{\frac{\theta }{2\pi }}\;e^{-\theta y^{2}/2}\;{\mathrm{He}}_{r}(\sqrt{\theta }y),\;\lambda_{r}=-\theta r,\;r\in N_{0}.$$

Importantly, up to normalization one has *ψ*_*r*+1_=*ψ*_*r*_′.

Now in the general case we know *ψ*_0_, which is the invariant density, and λ_0_=0, but we do not know any of the other eigenfunctions or eigenvalues. However, let us assume that the first eigenfunction is approximately *ψ*_0_′, and write ${\hat{\psi }}_{1}=\psi_{0}^{\prime }$ for this approximated eigenfunction. Then

$$L^{†}{\hat{\psi }}_{1}=\psi_{0}^{\prime \prime \prime }-{\left(A\psi_{0}^{\prime }\right)}^{\prime }={\left(A^{\prime }\psi_{0}\right)}^{\prime }.$$

Let us assume this to be roughly $\lambda_{1}{\hat{\psi }}_{1}$, i.e. λ_1_*ψ*_0_′. Then integrating once, we have *A*′*ψ*_0_≈λ_1_*ψ*_0_, and then integration from −∞ to ∞ gives

3.8 $$\lambda_{1}\approx \langle A^{\prime }\rangle \equiv \int_{-\infty }^{\infty }A^{\prime }(y)f_{Y}(\infty ,y)\;dy=-\int_{-\infty }^{\infty }\frac{f_{Y}^{\prime }{\left(\infty ,y\right)}^{2}}{f_{Y}(\infty ,y)}\;dy<0;$$

given that in the OU case λ_1_=−*θ*, we use

3.9 $$\theta =-\langle A^{\prime }\rangle >0.$$

This has considerable intuitive appeal, as 〈−*A*′〉 is, in a sense, the average rate of mean reversion, and by construction is necessarily positive. Of the ‘OU deformations’, it is easily calculated in two of the four cases, but (1.5) requires an integral, to which we provide the first term in a Padé approximation:

3.10 $$(1.5):\;\langle -A^{\prime }\rangle \approx \frac{\nu -1}{\nu +1/2};\;(1.6):\;\langle -A^{\prime }\rangle =\frac{\nu -2}{\nu +1};\;(1.7):\;\langle -A^{\prime }\rangle =\frac{\nu^{2}}{\nu^{2}-1}.$$

In the double-well example,

3.11 $$(1.9):\;\langle -A^{\prime }\rangle =\frac{7-8\pi^{1/2}\;e^{1/4}\Phi (-1/\sqrt{2})}{3}\approx 0.878$$

with *Φ* the cumulative Normal distribution function.

The above discussion gives an unambiguous prescription for *θ*, but it should not be thought that the value is critical. For example, one can expand the OU model *A*(*y*)=−θ*y using any *θ*>0, thereby picking up a series of correction terms that arise from the inequality of *θ* and θ*. It is easily verified that the resulting series is convergent for |1−*q*|<1, i.e. all *τ*≥0.

### (c) Initial results; the remainder term

**Figure 5.**
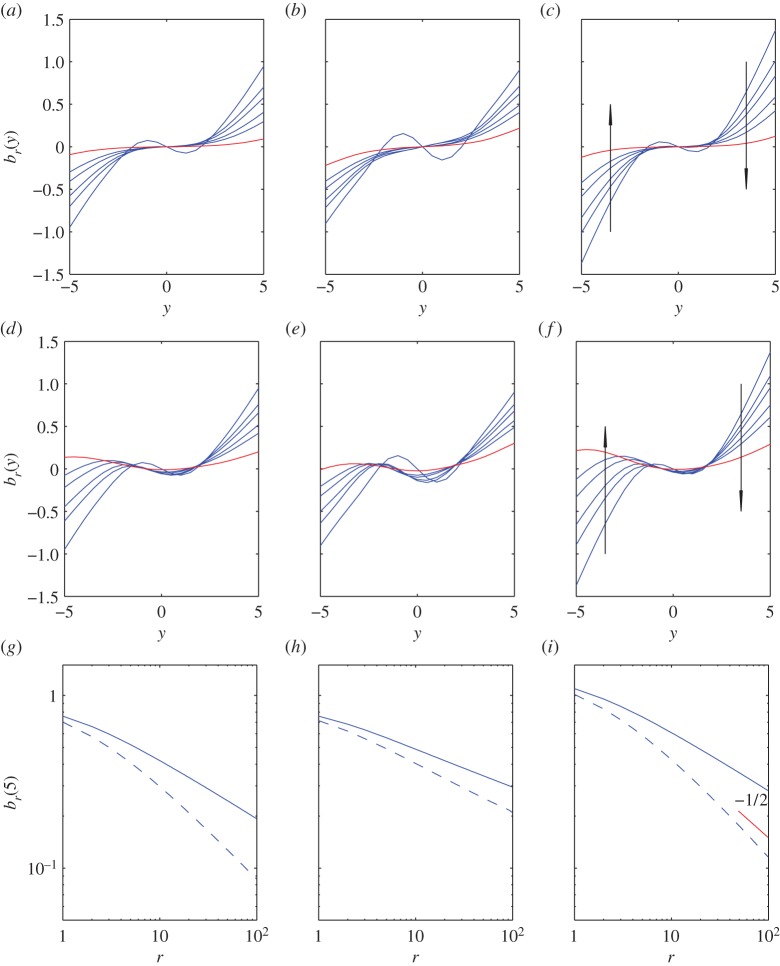
Panels (*a*–*c*) show *b*_*r*_(*y*) versus *y* for *r*=0,1,2,5,10,90 (arrows in (*c*) and (*f*) show the direction of increasing *r*) for the three models: (*a*) (1.5), (*b*) (1.6), (*c*) (1.7), with *Y*_0_=0. (*d*–*f*) are the same, but now with initial condition *Y*_0_=−2. Panels (*g*–*i*) show the variation of *b*_*r*_(5) versus *r* for *Y*_0_=0 (dashed) and *Y*_0_=−2 (solid). Panels are for (*g*) (1.5), (*h*) (1.6), (*i*) (1.7) and the slope $-\frac{1}{2}$ is shown in panel (*i*) for reference.

For a numerical demonstration, we set $\hat{\gamma }=\frac{1}{2}$, i.e. *ν*=5. Figure 5 shows the functions *b*_*r*_(*y*) for *r*=0,1,2,… for each of the three models ((1.5)–(1.7)), and with *Y*_0_=0,−2 in each. (From (3.10) we have *θ*=0.727,0.50,1.04, respectively.)

Apparently, the partial sums converge at power law in *r*, i.e. *b*_*r*_(*y*)∝*r*^−λ^, as is corroborated by figure 5*c*. This is consistent with *h*_*Y*_ having a singularity of the form *q*^λ^ at *q*=0, which in turn implies exponential decay in *t* as t→∞ (which seems reasonable, given our previous discussion on spectral theory). There is no universal scaling law, so λ depends on the problem at hand.

An estimate of λ, even if rudimentary, can allow us to estimate the discarded part of the summation, at least to the extent that we get a better estimate than assuming it to be zero. Using the result given in appendix A, the assumption *b*_*r*_(*y*)∝*r*^−λ^ leads to the approximation

3.12 $$q\sum_{r=N}^{\infty }{\left(1-q\right)}^{r}b_{r}(y)\approx \frac{{\left(1-q\right)}^{N}q^{\lambda }{\left(N-1\right)}^{\lambda }}{{\left(1+q(N-1)\right)}^{\lambda }}b_{N-1}(y).$$

In the examples we have investigated, we have found λ to lie between 0.2 and 0.6. One can use an empirical estimate, but we suggest using $\lambda =\frac{1}{2}$ throughout—a principle to which we will have recourse later—and applying (3.12) when N≳5.

We have also computed the partial sums *h*_*Y*_ with the solution obtained from a PDE solver to satisfy ourselves that they converge to the correct result. Wherever we refer to a PDE solver, we have used the method of lines with finite differences in space and DASSL (see [29]) as the time solver; this is a well-established, highly effective, and often used approach for numerically solving potentially highly nonlinear and stiff diffusion-like equations in fluid mechanics and elsewhere [30]. To compute *h*_*Y*_, we compute *g*_*Y*_ and then take the logarithmic derivative, rather than solving the nonlinear PDE (2.2) directly.

### (d) Derivation of the normalizing factor

We now know *h*_*Y*_(*τ*,*y*), but this only allows us to reconstruct *g*_*Y*_(*τ*,*y*) up to an arbitrary multiplicative time-dependent factor *n*_*Y*_(*τ*) say, which we must now obtain:

3.13 $$g_{Y}(\tau ,y)=n_{Y}(\tau )\exp\left(-\int_{Y_{0}}^{y}h_{Y}(\tau ,y^{\prime })\;dy^{\prime }\right).$$

(The lower limit of the integral is arbitrary, and taken as *Y*_0_ for convenience.) Inserting this into (2.1) gives a first-order linear differential equation for *n*_*Y*_:

$$\frac{1}{n_{Y}}\frac{\partial n_{Y}}{\partial \tau }=\int_{Y_{0}}^{y}\frac{\partial h_{Y}}{\partial \tau }\;dy-Ah_{Y}-\frac{\partial h_{Y}}{\partial y}+h_{Y}^{2}$$

and its solution must be, as $n_{Y}(\infty )=1$,

$$n_{Y}(\tau )=\exp\left\{\int_{\tau }^{\infty }\left(-\int_{Y_{0}}^{y}\frac{\partial h_{Y}}{\partial \tau }\;dy+Ah_{Y}+\frac{\partial h_{Y}}{\partial y}-h_{Y}^{2}\right)d\tau \right\}.$$

Note that the r.h.s. seems to depend on *y*, but does not actually do so, because *h*_*Y*_ obeys (2.2). Thus any *y* can be chosen, and using the same value as the lower limit of the *y*-integral causes the ∫dy term to vanish:

3.14 $$n_{Y}(\tau )=\exp\left\{\int_{\tau }^{\infty }{\left(Ah_{Y}+\frac{\partial h_{Y}}{\partial y}-h_{Y}^{2})\right|}_{y=Y_{0}}\;d\tau \right\}.$$

Substituting (3.1) into (3.14) and performing the time integral (note d*τ*=−d*q*/2*θq*) gives

3.15 nY(τ)=11−qexp{−12θ∑r=0N−1B(2,r+1;q)∑j=0rβjβr−j+ρN(τ)q−q1−qθY02−Y0(A(Y0)+θY0)ln⁡(1+q)+12θ∑r=0N−1B(1,r+1;q)(A(Y0)βr+βr′)−∑r=0N−1(B(2,r;q)−B(32,r;q))Y0βr−12θ∑r=0N−1B(2,r+1;q)∑j=0rβjβr−j+ρN(τ)},

where the coefficients (*β*_*r*_), (*β*′_*r*_) are defined by

3.16 $$\beta_{r}=b_{r}(Y_{0}\;|\;Y_{0}),\;\beta_{r}^{\prime }=b_{r}^{\prime }(Y_{0}\;|\;Y_{0}).$$

Remember, as emphasized by the ⋅ | *Y*_0_ notation in (3.16), that the functions (*b*_*r*_), for *r*≥1, depend parametrically on *Y*_0_. Thus, if *Y*_0_ is altered then one must recalculate all the (*b*_*r*_) from *r*=1 upwards, using (3.7), for (3.15) to remain correct: it is insufficient to keep the same (*b*_*r*_) and evaluate them at a different point. The symbol B(*a*,*b*;*q*) denotes the incomplete Beta function,

$$B(a,r;q)=\int_{0}^{q}p^{a-1}{\left(1-p\right)}^{r-1}\;dp.$$

Note that

$$B(a,1;q)=\frac{q^{a}}{a},\;B(1,r;q)=\frac{1-{\left(1-q\right)}^{r}}{r}$$

and

$$B(2,r;q)=\frac{1-{\left(1-q\right)}^{r}}{r}-\frac{1-{\left(1-q\right)}^{r+1}}{r+1},$$

whereas $B(\frac{3}{2},r;q)$ requires a recurrence:

$$B\left(\frac{3}{2},r;q\right)=\frac{2}{2r+1}q^{3/2}{\left(1-q\right)}^{r-1}+\frac{2(r-1)}{2r+1}B\left(\frac{3}{2},r-1;q\right).$$

In the OU case, *A*(*y*)≡−*θy*, the only effective terms in (3.15) are the (1−*q*)^−1/2^ prefactor and the first exponential term, with the $Y_{0}^{2}$ in it; this is as it should be. In the special case where *A* is an odd function, as it is with all of our examples, and *Y*_0_=0 also, this simplifies to

3.17 $$\text{[}A\text{ odd]}\;n_{Y}(\tau )=\frac{1}{\sqrt{1-q}}\exp\left\{\frac{1}{2\theta }\sum_{r=0}^{N-1}\frac{1-{\left(1-q\right)}^{r+1}}{r+1}\beta_{r}^{\prime }{|}_{Y_{0}=0}+\rho_{N}(\tau )\right\}.$$

Now (3.15) and its special case (3.17) are rather slowly convergent for small *τ*, i.e. *q*→1. This difficulty can in fact be finessed, as we now show. Multiply both sides in (3.13) by $f_{Y}(\infty ,y)$, and set *y*=*Y*_0_ and *τ*→0, getting

fY(τ,Y0)∼τ→0fY(∞,Y0)1−qexp{12θ∑r=0N−1B(2,r+1)∑j=0rβjβr−j−∑r=0N−1(B(2,r)−B(32,r))∗βr+ρN(0)12θY02−Y0(A(Y0)+θY0)ln⁡2+12θ∑r=0N−1B(1,r+1)(A(Y0)βr+βr′)−12θ∑r=0N−1B(2,r+1)∑j=0rβjβr−j−∑r=0N−1(B(2,r)−B(32,r))∗βr+ρN(0)}.

The * symbol signifies the following: when *r*=0 the Beta function B(*a*,*r*) is undefined, but $B(2,r)-B(\frac{3}{2},r)$ is taken to mean the value of $B(2,r;q)-B(\frac{3}{2},r;q)$ in the limit *q*↗1, which is well defined and in fact equals 1−2ln⁡2. But by direct analysis of the FP equation, we find

$$f_{Y}(\tau ,Y_{0})\overset{\tau \to 0}{∼}\frac{1}{\sqrt{4\pi \tau }}\overset{\tau \to 0}{∼}\frac{1}{\sqrt{2\pi (1-q)/\theta }}.$$

By comparison of these two results, we are able to establish two things, or, rather, one thing that can be used in two ways. First, we have an infinite product representation of the invariant density at the point *Y*_0_:

3.18 fY(∞,Y0)=e−θY02/22π/θexp{∑r=0N−1(B(2,r)−B(32,r))∗Y0βr+12θ∑r=0N−1B(2,r+1)∑j=0rβjβr−j−ρN(0)Y0(A(Y0)+θY0)ln⁡2−12θ∑r=0N−1B(1,r+1)(A(Y0)βr+βr′)+∑r=0N−1(B(2,r)−B(32,r))∗Y0βr+12θ∑r=0N−1B(2,r+1)∑j=0rβjβr−j−ρN(0)}.

As we said above, altering *Y*_0_ requires a complete recalculation of the (*β*_*r*_). But in fact only one point is needed to determine $f_{Y}(\infty ,y)$ for all *y*, since

$$f_{Y}(\infty ,y)=f_{Y}(\infty ,Y_{0})\exp\left(\int_{Y_{0}}^{y}A(y)\;dy\right).$$

In the symmetrical case where *A* is an odd function, it is obvious to choose *Y*_0_=0, and then (3.18) simplifies to

3.19 $$\text{[}A\text{ odd]}\;f_{Y}(\infty ,0)=\frac{1}{\sqrt{2\pi /\theta }}\exp\left(-\frac{1}{2\theta }\sum_{r=0}^{N-1}\frac{\beta_{r}^{\prime }{|}_{Y_{0}=0}}{r+1}-\rho_{N}(0)\right).$$

However, $f_{Y}(\infty ,y)$ is often known, as we said earlier with (1.5)–(1.7), or else can be calculated as an exponential integral followed by normalization to unit probability mass. The second idea, therefore, is to reverse the previous logic and use $f_{Y}(\infty ,y)$ to obtain *ρ*_*N*_(0):

3.20 $$\begin{array}{rl} \rho_{N}(0) & =\ln\left(\frac{e^{-\theta Y_{0}^{2}/2}/\sqrt{2\pi /\theta }}{f_{Y}(\infty ,Y_{0})}\right)+Y_{0}(A(Y_{0})+\theta Y_{0})\ln2-\frac{1}{2\theta }\sum_{r=0}^{N-1}B(1,r+1)(A(Y_{0})\beta_{r}+\beta_{r}^{\prime })\\ & \;+\sum_{r=0}^{N-1}{\left(B(2,r)-B\left(\frac{3}{2},r\right)\right)}_{*}Y_{0}\beta_{r}+\frac{1}{2\theta }\sum_{r=0}^{N-1}B(2,r+1)\sum_{j=0}^{r}\beta_{j}\beta_{r-j}; \end{array}$$

or, when *A* is odd,

3.21 $$[A\;\mathrm{odd}]\;\rho_{N}(0)=\ln\left(\frac{e^{-\theta Y_{0}^{2}/2}/\sqrt{2\pi /\theta }}{f_{Y}(\infty ,Y_{0})}\right)+Y_{0}(A(Y_{0})+\theta Y_{0})\ln2-\frac{1}{2\theta }\sum_{r=0}^{N-1}B(1,r+1)\beta_{r}^{\prime }.$$

The first term in either of the above is the logarithm of the quotient of the Normal distribution (mean 0, variance 1/*θ*) and the true invariant density.

We can use this (exact) expression for *ρ*_*N*_(0) to sharpen the approximation in (3.15), thereby making it match at *τ*=0; recall that it already does so at τ=∞. Judging from (3.17) and the discussion surrounding (3.12), *β*_*r*_′=*O*(*r*^−λ^) as r→∞ for some positive λ, and the expression $\sum_{r=1}^{\infty }r^{-\lambda -1}{\left(1-q\right)}^{r}$ defines an analytic function of *q* that is of order *q*^λ^ as *q*↘0, by the Tauberian theorem. Accordingly, we are motivated to write

3.22 $$\rho_{N}(\tau )=\rho_{N}^{♯}(\tau )+q^{\lambda }\rho_{N}(0).$$

Thus, $\rho_{N}^{♯}(0)=\rho_{N}^{♯}(\infty )=0$ for all *N*. As we can easily compute *ρ*_*N*_(0), we reduce the truncation error in (3.15) from *ρ*_*N*_(*τ*) to $\rho_{N}^{♯}(\tau )$. As *any* λ>0 will exactly remove the truncation error at *τ*=0, we are free to choose $\lambda =\frac{1}{2}$ throughout, as previously intimated.

### (e) Leading-order expansion

We are now ready to put everything together, and in the first instance we use no correction terms, thereby ignoring all the (*b*_*r*_). We have in this approximation, by (3.1),

$$\exp\left(-\int_{Y_{0}}^{y}h_{Y}(\tau ,y^{\prime })\;dy^{\prime }\right)\overset{N=0}{\approx }\exp\left(\frac{-(1/2)\theta q(y^{2}-Y_{0}^{2})+\theta \sqrt{q}Y_{0}(y-Y_{0})}{1-q}\right);$$

and by (3.15), (3.20) and (3.22),

$$\begin{array}{rl} n_{Y}(\tau ) & \overset{N=0}{=}\frac{1}{\sqrt{1-q}}\exp\left(\frac{\sqrt{q}-q}{1-q}\theta Y_{0}^{2}-Y_{0}(A(Y_{0})+\theta Y_{0})\ln(1+\sqrt{q})+q^{\lambda }\rho_{0}(0)\right)\\ \rho_{0}(0) & \approx \ln\left(\frac{e^{-\theta Y_{0}^{2}/2}/\sqrt{2\pi /\theta }}{f_{Y}(\infty ,Y_{0})}\right)+Y_{0}(A(Y_{0})+\theta Y_{0})\ln2. \end{array}$$

Multiplying these two to get *g*_*Y*_(*τ*,*y*), and then by $f_{Y}(\infty ,y)$, gives us what we are after:

3.23 $$\begin{array}{rl} f_{Y}(\tau ,y) & \overset{N=0}{\approx }\frac{1}{\sqrt{1-q}}{\left(\frac{e^{-\theta Y_{0}^{2}/2}/\sqrt{2\pi /\theta }}{f_{Y}(\infty ,Y_{0})}\right)}^{q^{\lambda }}\exp\left(Y_{0}(A(Y_{0})+\theta Y_{0})\ln\left(\frac{2^{q^{\lambda }}}{1+\sqrt{q}}\right)\right)\\ & \;\times f_{Y}(\infty ,y)\exp\left(\frac{-(1/2)\theta q{\left(y-Y_{0}\right)}^{2}+\theta (\sqrt{q}-q)\;Y_{0}y}{1-q}\right). \end{array}$$

In effect, this is the invariant density multiplied by a Gaussian of time-dependent width and centre; we recall that *θ* comes from (3.9) and (3.10), and we are standardizing on $\lambda =\frac{1}{2}$.

**Figure 6.**
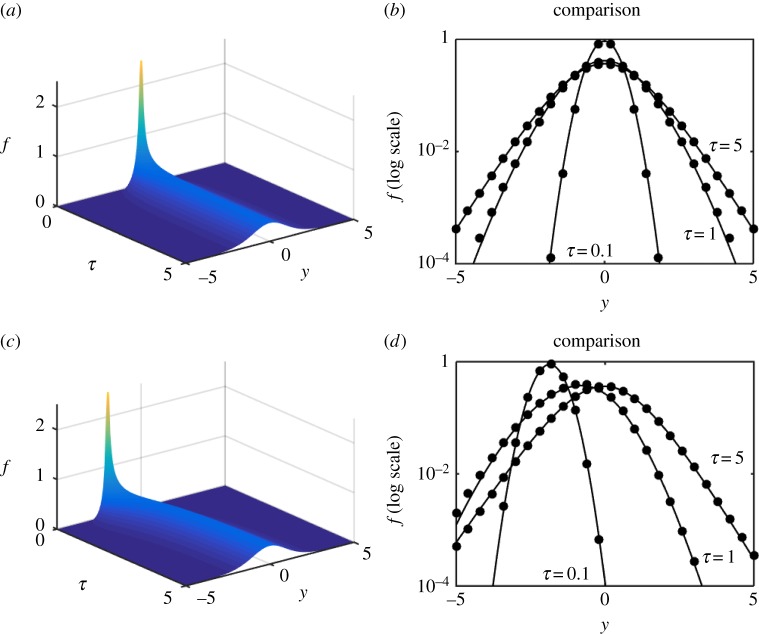
Numerics and leading-order expansion compared for the model (1.5). Panels (*a*,*b*) are for *Y*_0_=0 and (*c*,*d*) are for *Y*_0_=−2. (*a*,*c*) The numerical solution for *f*_*Y*_(*τ*,*y*) and (*b*,*d*) the numerical solution (solid) and the leading-order (*N*=0) expansion (dots) for *τ*=0.1,1,5.

**Figure 7.**
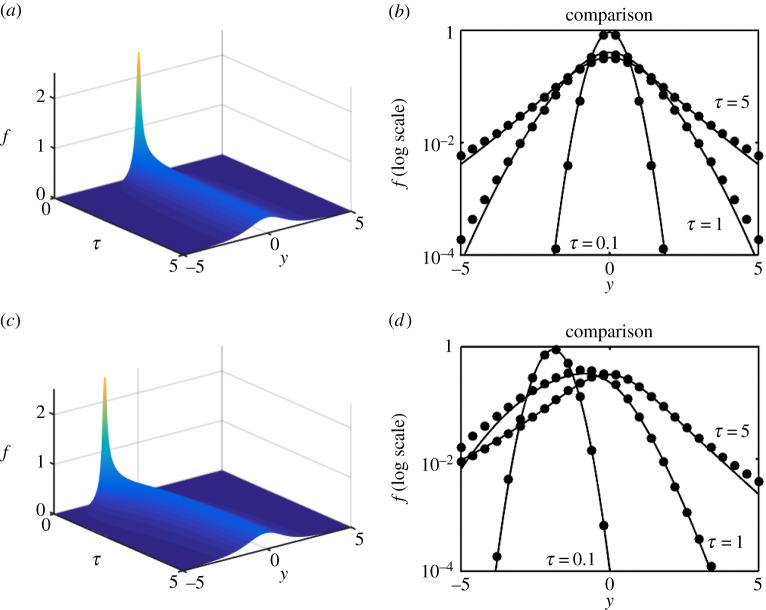
Numerics and leading-order expansion compared for the model (1.6). Panels (*a*,*b*) are for *Y*_0_=0 and (*c*,*d*) are for *Y*_0_=−2. (*a*,*c*) The numerical solution for *f*_*Y*_(*τ*,*y*) and (*b*,*d*) the numerical solution (solid) and the leading-order (*N*=0) expansion (dots) for *τ*=0.1,1,5.

**Figure 8.**
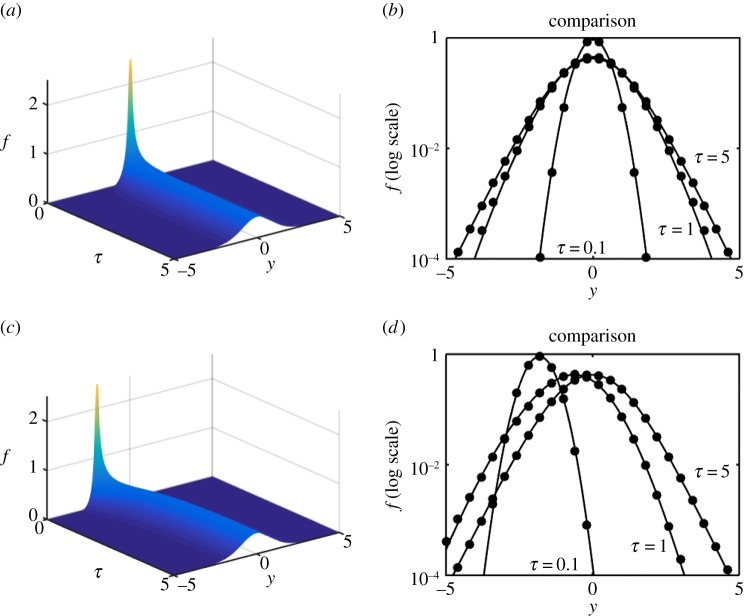
Numerics and leading-order expansion compared for the model (1.7). Panels (*a*,*b*) are for *Y*_0_=0 and (*c*,*d*) are for *Y*_0_=−2. (*a*,*c*) The numerical solution for *f*_*Y*_(*τ*,*y*) and (*b*,*d*) the numerical solution (solid) and the leading-order (*N*=0) expansion (dots) for *τ*=0.1,1,5.

**Figure 9.**
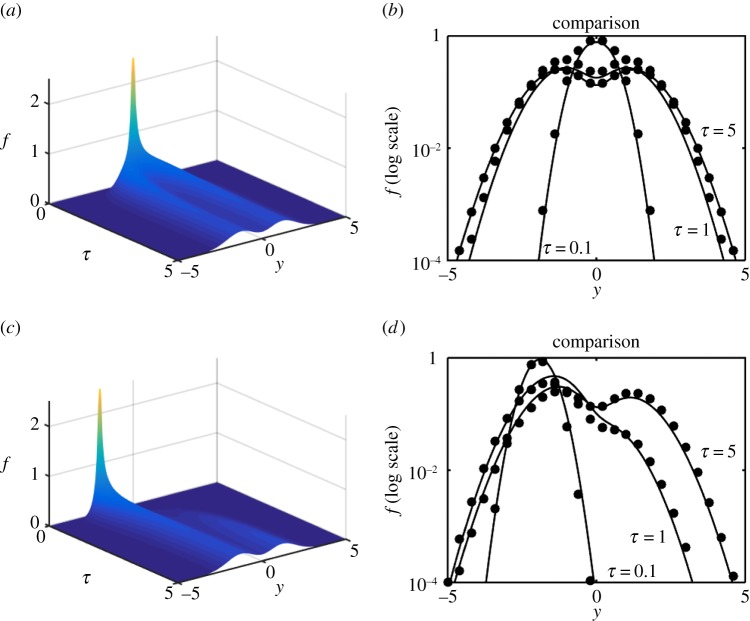
Numerics and leading-order expansion compared for the double-well example of equation (1.9). Panels (*a*,*b*) are for *Y*_0_=0 and (*c*,*d*) are for *Y*_0_=−2. (*a*,*c*) The numerical solution for *f*_*Y*_(*τ*,*y*) and (*b*,*d*) the numerical solution (solid) and the leading-order (*N*=0) expansion (dots) for *τ*=0.1,1,5.

We are now in a position to explore the efficacy of the expansion scheme versus full numerical solutions and we proceed to do so in figures 6–8. Each shows a numerical simulation of the full PDE, using the same parameters as in §3*c*, i.e. $\hat{\gamma }=\frac{1}{2}$, for *Y*_0_=0,−2; the shift in the source position illustrates that, in each case, the solution drifts back to the origin as it simultaneously diffuses outward. In each case, we illustrate for *f*, on log axes to accentuate any error, the numerical solution of the PDE for *τ*=0.1,1,5 versus the *N*=0 (just the leading-order) solution (3.23). The solutions are virtually indistinguishable. As a more stringent, and demanding, test upon the methodology the double-well example (1.9) is also evaluated both numerically and via the expansion; the results shown in figure 9 are again remarkably accurate, particularly considering that it is just the *N*=0 approximation that is shown.

### (f) Higher order development

**Figure 10.**
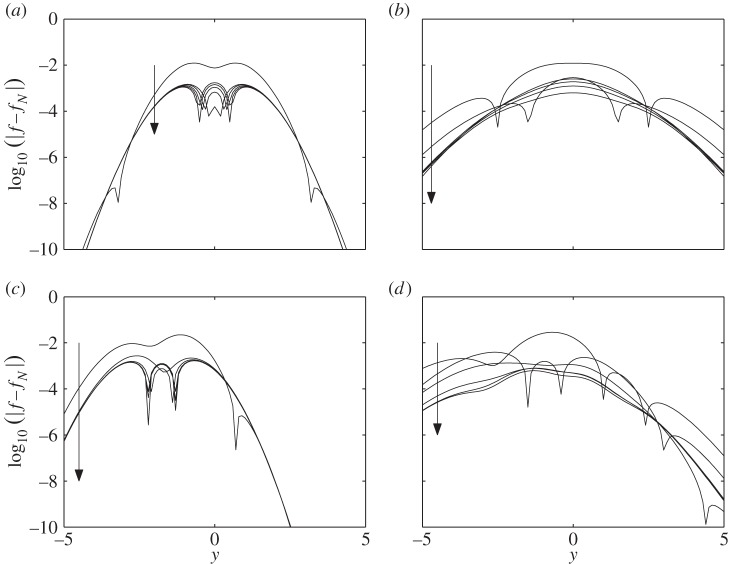
The log_10_ difference between the numerical solution and the analytic approximations to (1.5) for different truncations of *N*. Each panel shows this difference for *N*=0,2,5,10,20,50 versus *y* with the arrow showing the direction of increasing *N*. Panels (*a*,*b*) are for *Y*_0_=0 at *t*=0.5 and 2 respectively, and (*c*,*d*) are identical except that *Y*_0_=−2.

We can, however, pursue the approximation to higher order in an attempt to squeeze the error down further. This we illustrate by plotting the difference between the numerical solution for the density and the approximation (summarized by equations (3.1), (3.13), (3.15), (3.20) and (3.22)) in figure 10. The numerical solution is evaluated using a highly accurate implicit time solver and uses central differences that are accurate to *O*(*δy*^3^)∼*O*(10^−6^), where *δy* is the gridspacing that is 10^−2^ in the simulations shown; thus the observed difference of *O*(10^−6^) is to be expected. Figure 10 shows the *N*=0 approximation together with increasing values of *N* (2,5,10,20,50) and it appears that the error converges to zero as N→∞. For reasons of space, we just show the results for model (1.5), but the picture is similar for the other cases.

The integrals in (3.7) are algebraically intractable in general, and by way of numerical techniques we suggest the use of Chebyshev approximation. Set up an interval $\left[y_{\min},y_{\max}\right]$, approximate *b*_*r*_ at the Chebyshev nodes (*y*_*k*_), which allows its derivatives to be evaluated at any point as a function of the expansion coefficients [11]; then evaluate at each *k* the integral (3.7) by Gauss–Legendre quadrature (which, apart from the term containing *A*, will be exact provided the quadrature order is $\geq \frac{1}{2}r^{*}+m-\frac{1}{2}$, where *m* is the degree of the Chebyshev approximation,^2^ and r* is the highest value of *r* desired in (3.7)), and use these values *b*_*r*_(*y*_*k*_) as the samples of an interpolating Chebyshev approximation. The recurrence is initialized by approximating *b*_0_. Incidentally, this method was used to generate the results in figure 5 using degree 21 on the interval [−10,10]. Note that *y*↦*h*_*Y*_(*τ*,*y*) is approximated as a polynomial, for each *τ*; the integral in (3.13) can be done by Gaussian quadratures again or directly from the Chebyshev expansion of the (*b*_*r*_).

### (g) Divergent (unstable) diffusions

An intriguing question is whether the methods described here also work for ‘unstable’ (non-reverting) diffusions, i.e. those with no invariant density. Hongler & Desai [31] point out that the so-called repulsive Wong model admits a closed-form solution in one isolated case. This is

$$dY_{t}=2\alpha \tanh y\;dt+\sqrt{2}\;dW_{t},$$

whose FP equation solves for *α*=1 as

$$f_{Y}(t,y)=\frac{e^{-{\left(y-(Y_{0}+2t)\right)}^{2}/4t}\;e^{Y_{0}}+e^{-{\left(y-(Y_{0}-2t)\right)}^{2}/4t}\;e^{-Y_{0}}}{4\sqrt{\pi t}\cosh Y_{0}}=\frac{e^{-t}}{2\sqrt{\pi t}}\cdot e^{-{\left(y-Y_{0}\right)}^{2}/4t}\frac{\cosh y}{\cosh Y_{0}}.$$

Now the above diffusion is *not* reconcilable with (1.7), on account of the sign in the drift being wrong. Inasmuch as it is related to the OU process, one has to take (1.4) with negative mean reversion (*κ*=−1, *σ*=1, *γ*=1), and then transform by $X=\sinh\frac{1}{2}Y$. There is, of course, no invariant density. There is an unphysical steady-state solution to the FP equation, which is $\propto \cosh^{2}y$. However, division of *f*_*Y*_ by this to get the solution to the adjoint forward equation does not give a solution that tends to 1 as t→∞, so the methods given in this paper, and in particular (3.23), fall apart. Note that the form of the solution is two diverging blobs of probability mass. This is important: whereas mean-reverting diffusions do look like the standard OU process, allowing the solution to the FP equation to be expanded around the solution of the OU process, divergent ones may exhibit structural features particular to themselves and cannot be regarded in the same way.

That said, the quantity $(-\partial /\partial y)\ln\;f_{Y}$ is simple,

$$-\frac{\partial \;}{\partial y}\ln f_{Y}(t,y)=\frac{y-Y_{0}}{2t}-\tanh y,$$

and it does have a large-time limit, −tanh⁡y. But this limit is *not* obtainable from the logarithmic derivative of the unphysical steady-state solution, as that is

$$-\frac{\partial \;}{\partial y}\ln\cosh^{2}y=-2\tanh y.$$

We may fairly state the following conclusions: (i) the expansion shown here is inapplicable to divergent diffusions; (ii) an alternative form of expansion of the logarithmic derivative of the density may well prove useful; and (iii) it is an interesting avenue for further research.

### (h) Convergence

The series (3.1) is derived as an expansion in the limit *t*→0. Thus, it may or may not be convergent in the classical sense, i.e. $\lim_{N\to \infty }R_{N}(\tau ,y)=0$ for each *τ*,*y*. For the examples we have considered, the empirical evidence is that *b*_*r*_(*y*) asymptotically decays at power order in *r* as r→∞, and this is sufficient for the purpose.

Clearly, some conditions must be obeyed by *A* for convergence to hold. While the *N*=0 approximation (3.23) is generally applicable, the usefulness of higher order terms is predicated on differentiability of *A*, because, as the order of the approximation (*N*) is increased, successively higher derivatives of *A* are invoked, on account of the *b*_*r*_′′ term. It seems likely that a necessary condition on *A* is that it be analytically continuable to the open strip |Im *y*|<*η*, for some *η*>0. What further conditions are required for *h*_*Y*_ to have the posited Laurent expansion (3.1) convergent in the punctured disc |*q*−1|<1 is a matter for further research.

We reiterate that, whether the series is classically convergent or not, the error is uniformly bounded in *τ*, by contrast with an expansion in powers of *τ*.

### (i) Fusion with existing techniques

We argue that it is possible to combine the best features of the series expansion shown here with those of classical methods of solving PDEs. If (3.1) is non-convergent, it will be impossible to obtain arbitrary accuracy (in general, this is a perennial problem with asymptotic expansions: they give excellent accuracy in certain regions but cannot achieve arbitrary accuracy at any given point)—and, even if it does converge, its convergence may be inconveniently slow. Classical techniques and the existence theorems that relate to them are not reliant on analyticity, but can potentially obtain arbitrary accuracy. (From a more general context: on a compact interval any continuous function may be approximated to arbitrary accuracy by a polynomial or a Fourier series—but neither constitutes any sort of power series expansion of the function, as it may well not be analytic. In approximation theory, one does not want to be forced to assume analyticity of the function being approximated.)

Consider an approximate solution *f*^♯^(*τ*,*y*), such as the *N*=0 approximation though any other *N* would do, and define the relative error *ψ*_*Y*_ via the substitution

$$f_{Y}(\tau ,y)=f_{Y}^{♯}(\tau ,y)\;e^{\psi_{Y}(\tau ,y)},$$

then writing down the PDE obeyed by *ψ*_*Y*_. As we have extracted the *τ*=0 and τ→∞ behaviour correctly in $f_{Y}^{♯}$, the initial condition is *ψ*_*Y*_≡0 (and we expect *ψ*_*Y*_ to vanish as τ→∞). The resulting equation for *ψ*_*Y*_, though nonlinear, can still be solved by grid-based methods or by spectral methods. But whereas, before, we said that spectral methods have difficulty coping with a singular initial condition, they are now being applied to the slowly varying function *ψ*_*Y*_, and so their performance will be much better. In essence, as far as numerics are concerned, all the hard work has been done by extracting $f_{Y}^{♯}$.

This idea has been used in different guises for many decades. For example in [21], it is common to see special functions approximated similarly. After studying the expansion's behaviour and/or singularities, apply an appropriate transformation to take these out and map the domain to [−1,1]. The transformed function, which by construction is slowly varying and properly behaved at the endpoints, is then ideally approximated as a Chebyshev series—and this is the kind of problem for which spectral methods are ideal.

## 4. Conclusion

We have shown how to expand the solution of the forward and backward equations of modified OU processes ‘around’ the OU case in a way that respects the characteristics of the problem. The method generalizes to other processes, and our conclusions are summarized as follows.

— It is more convenient to work in normalized coordinates, hence the transformation from *X* to *Y* and the time change from *t* to *τ*. By this transformation, we can assume that the volatility term is constant. Throughout, *q*=e^−2*θτ*^ so that *q*∈[0,1] and we expand in *q*(1−*q*)^*r*^. The suggested value of *θ* is that given by (3.9).
— The solution to the forward equation is given by $f_{Y}(\tau ,y)=g_{Y}(\tau ,y)f_{Y}(\infty ,y)$, where *g*_*Y*_ solves the adjoint forward equation (also known as the backward equation).
— The infinite product expansion of *g*_*Y*_ is given by (3.13) and (3.1). If only the logarithmic derivative (w.r.t. *y*) is needed, then this is just −*h*_*Y*_, so *n*_*Y*_ can be ignored.
— The functions *b*_*r*_ are obtained by the recurrence (3.5) and (3.7), using also (3.2).
— The function *n*_*Y*_ is given by (3.15), and the remainder term *ρ*_*N*_(*τ*) is decomposed by (3.22) into a part $\sqrt{q}\rho_{N}(0)$ that can be evaluated by (3.20) and a pared-down remainder term $\rho_{N}^{♯}$ which is dropped.
— The case with no correction terms, *N*=0, is given by (3.23).

In terms of computation, we have found that this method improves upon the use of a standard PDE solver. One reason for this is that, in common with spectral methods, it gives arbitrary spatial resolution, whereas to get a spatial resolution of *O*(*δx*) using a PDE solver with, say, standard finite differences typically requires a time step of *O*(*δx*)^2^. We have found that the approximation, with a handful of correction terms, typically represents an improvement in speed of a couple of orders of magnitude over the PDE solver; using *N*=0 is obviously even faster.

## 5. Further developments

This section discusses possible avenues for further research, which we think are numerous and varied.

— Multi-dimensional analogues. These would require the expansion of a multi-dimensional problem around its associated multi-dimensional OU ansatz.
— Time-varying analogues. In principle, the logarithmic derivative of the associated FP equation has a useful structure even when the coefficients of the underlying stochastic differential equation are time-varying, but it is less clear how to proceed as a steady-state solution can no longer be identified: thus one might have to work directly with the FP equation rather than its adjoint.
— If the process is strictly positive, as happens in certain financial models, then the natural ansatz is no longer the OU process, but instead the square-root process
  $$dY_{t}=(a-\kappa Y_{t})\;dt+\sigma \sqrt{Y_{t}}\;dW_{t},$$
  for which the FP equation has a known solution (e.g. [32]). This would allow such processes as
  $$dX_{t}=(a-\kappa X_{t})\;dt+\sigma \sqrt{X_{t}(1+\gamma X_{t})}\;dW_{t}$$
  to be analysed. A further development is when the process is bounded on both sides, such as
  $$dX_{t}=(a-\kappa X_{t})\;dt+\sigma \sqrt{1-X_{t}^{2}}\;dW_{t}.$$
— The representation of the solution to the forward equation arising from a Lévy process.
— Barrier problems. These fall into two broad categories. One possibility is to solve the FP equation with a delta-function initial condition, in the presence of one or more absorbing barriers. In that case, an infinite product solution can be thought of as a term corresponding to the initial condition, a set of terms that vanish on the boundary(ies), and the exponential of a remainder series. An alternative is to solve the backward equation, giving the density of the stopping-time conditionally on starting from some point *X*_0_. This would allow insight to be gained into such problems as the first passage time of an OU process through a barrier: no simple solution is available, but approximate analytical forms are known, and an infinite product expansion should allow the errors in these to be quantified and corrected in a sensible way.

## Supplementary Material

idx.dat. lennar.dat. butterfly.dat

## Footnotes

1 [12], p. 286: correspondence of P. Hagan, dated 7 February 2012.

2 One less than the order.

## Data accessibility

The datasets supporting this article have been uploaded as part of the electronic supplementary material.

## Appendix A. Remainder term approximation

Let

$$S_{q,N}=q\sum_{r=N}^{\infty }{\left(1-q\right)}^{r}r^{-\lambda }.$$

We derive the approximation, in the limits *q*→0, N→∞:

$$S_{q,N}\approx \frac{{\left(1-q\right)}^{N}q^{\lambda }}{{\left(1+q(N-1)\right)}^{\lambda }}.$$

Following an argument of Riemann employed in a similar context, we use the identity

$$r^{-\lambda }=\frac{1}{\Gamma (\lambda )}\int_{0}^{\infty }x^{\lambda -1}\;e^{-rx}\;dx;$$

summing over *r*, we find, on writing *ξ*=*Nx*,

$$S_{q,N}=\frac{q{\left(1-q\right)}^{N}}{N^{\lambda }\Gamma (\lambda )}\int_{0}^{\infty }\frac{\xi^{\lambda -1}\;e^{-\xi }}{1-(1-q)\;e^{-\xi /N}}\;d\xi .$$

It is necessary to approximate the term in the denominator for ξ≲1, and matching the function and its first derivative at 0 gives

$$\frac{1}{1-(1-q)\;e^{-\xi /N}}\approx q^{-1}e^{-(1-q)\xi /Nq};$$

this allows the integral to be performed, with the desired result.
